# Congrès International de Médecine

**Published:** 1899-09

**Authors:** 


### Congrès International de Médecine Professionelle et de Déontologie Médicale.

In connection with the Exposition Universelle in Paris, it is proposed to hold an International Medical Congress from the 23rd to the 28th of July, 1900. The members alone will have the right to take part in the discussions, and a fee of 15 francs will be required. The Editor of this *Journal* will be glad to receive the names of any persons in this district who may wish to be enrolled.

